# Personalized eye protection for head CT organ‐based tube current modulation: A deep learning approach to derive 3D eyeball models from a single‐view topogram

**DOI:** 10.1002/acm2.70665

**Published:** 2026-06-23

**Authors:** Xiaolin Meng, Lei Zhu, Shenghao Chen, Manhua Liu, Yang Wang

**Affiliations:** ^1^ School of Electronic Information and Electrical Engineering Shanghai Jiao Tong University Shanghai China; ^2^ Healthcare Advanced Algorithm Department of HSW BU Shanghai United Imaging Healthcare Co., Ltd Shanghai P.R. China; ^3^ Correction Algorithm Department of CT BU Shanghai United Imaging Healthcare Co., Ltd Shanghai P.R. China; ^4^ MoE Key Laboratory of Artificial Intelligence, AI Institute Shanghai Jiao Tong University Shanghai China; ^5^ Department of Radiology Zhujiang Hospital of Southern Medical University Guangzhou China; ^6^ Department of Radiology, Shanghai Tenth People's Hospital, Tongji University School of Medicine Shanghai China

**Keywords:** deep learning, eyeball model generation, organ‐based tube current modulation (OBTCM), radiation protection, topogram

## Abstract

**Background:**

The lens of the eye is highly radiosensitive, yet personalized shielding during head CT remains challenging due to the lack of a rapid, pre‐scan localization method.

**Purpose:**

To develop and validate a deep learning solution that enables automated, patient‐specific eye protection by generating a precise 3D eyeball model directly from a single‐view topogram.

**Methods:**

Our two‐stage approach combines an advanced data simulation pipeline—which generates realistic training topograms from digitally reconstructed radiographs (DRRs) using a table‐movement‐aware model and CycleGAN‐based stylization—with a dedicated generative network (EyeGen‐Net). The model was trained on 400 synthetic and validated on 100 real clinical samples.

**Results:**

EyeGen‐Net achieved a Dice Similarity Coefficient of 0.79 ± 0.08, a Hausdorff Distance of 5.40 ± 1.57 mm, and an Average Surface Distance of 1.84 ± 0.65 mm against expert segmentations. Crucially, phantom validation demonstrated that the derived 3D model facilitates organ‐based tube current modulation (OBTCM), yielding an approximate 30% reduction in lens dose across different scanning modes without compromising diagnostic image quality.

**Conclusions:**

This work provides a practical, automated pathway for implementing personalized radioprotection in routine head CT, aligning with the ALARA (As Low As Reasonably Achievable) principle.

## 1 INTRODUCTION

Computed tomography (CT) is indispensable in modern diagnostic imaging.^1^ However, the associated ionizing radiation exposure necessitates stringent protection measures, particularly for radiosensitive organs such as the eye lens. Radiation‐induced ocular damage, including cataracts and macular degeneration, can significantly impair vision.^2^, ^3^, ^4^ Furthermore, cumulative exposure increases lifetime cancer risk.^5^, ^6^, ^7^ These risks are especially pronounced in pediatric and young adult patients due to their heightened tissue radiosensitivity and longer life expectancy for potential effects to manifest.^8^, ^9^

The sensitivity of the ocular lens to radiation is well established. In the context of patient protection during diagnostic head CT, recent studies indicate that acute exposure can induce lens opacities at thresholds of approximately 0.5 Gy, with 90–95% confidence intervals including zero.^10^ These findings underscore the need for effective, personalized eye protection in routine CT.

Organ‐based tube current modulation (OBTCM) is a key technology for targeted dose reduction in modern CT.^11^, ^12^ It aims to minimize dose to critical organs while preserving diagnostic image quality. Current strategies to achieve personalized OBTCM often rely on topograms (scout scans). These strategies can be broadly categorized into two paradigms. The first, and more common, approach utilizes fixed angular sectors (e.g., a 90° to 180° anterior arc) where tube current is uniformly reduced.^13^, ^14^, ^15^ While straightforward to implement, this method cannot account for individual anatomical variation, potentially leading to either incomplete protection of the eyes or unnecessary degradation of image quality in noncritical regions. This twofold limitation was corroborated by our analysis of 100 clinical cases; detailed statistics are provided in Table S1.

The second, more advanced paradigm aims to create patient‐specific protection zones. This is achieved by generating synthetic CT images from topograms to derive precise 3D organ masks for dose modulation. For instance, deep learning algorithms have been developed to generate synthetic CTs from dual‐view topograms for organ‐based risk minimization.^16^, ^17^ Other workflows use topograms and prior information to create synthetic CTs for optimizing tube current distribution.^18^ While promising, these methods typically require dual‐view topograms, which are not always acquired in routine clinical protocols. More importantly, detailed technical disclosures for generating accurate masks for small, critical structures like the eyeball directly from a single‐view are notably lacking in the literature.

Consequently, a significant gap exists between the ideal of personalized protection and clinical practicality. The critical task of generating a precise 3D eyeball model from the single‐view topogram—the cornerstone of most routine head CT scans—remains unaddressed. This study aims to bridge this gap by introducing a novel, end‐to‐end deep learning framework. Our work systematically tackles the two primary challenges in this field: the scarcity of perfectly aligned topogram‐CT training pairs and the difficulty of segmenting small structures.

The main contributions of this work are threefold: (1) We introduce an advanced data simulation pipeline that generates highly realistic synthetic single‐view topograms, effectively overcoming clinical data scarcity. (2) We present the first detailed disclosure of a dedicated deep learning network (EyeGen‐Net) designed for accurate 3D eyeball mask generation from a single 2D topogram. (3) We provide comprehensive validation, including a phantom study that quantitatively assesses the radiation dose reduction and image quality impact of the enabled OBTCM.

## 2 METHODS

### 2.1 Data simulation and preparation

In this study, a “single‐view topogram” refers to the single planar projection image (typically anteroposterior or lateral) routinely acquired prior to diagnostic CT scanning for patient positioning and scan range planning. This represents the most common scanning mode in clinical head CT examinations. Unlike dual‐view topograms, which require acquisition from two angles, single‐view topograms have shorter acquisition time, lower radiation exposure, and are the default setting in most routine head CT protocols.

A paired dataset of topograms and corresponding CT volumes with annotated eyeball masks is essential for training. As no such public dataset exists and collecting a large number of perfectly aligned clinical pairs is prohibitively expensive—due to inevitable patient motion between the topogram and diagnostic scan—we developed a robust data simulation pipeline. We utilized 400 real CT volumes to generate synthetic topograms for training. An additional 100 clinically acquired, anonymized paired topogram‐CT datasets were collected from a single institution for validation. All clinical data usage was approved by the institutional review board, with patient consent waived for this retrospective analysis.

#### 2.1.1 Conventional and proposed digitally reconstructed radiograph (DRR) generation

**FIGURE 1:**
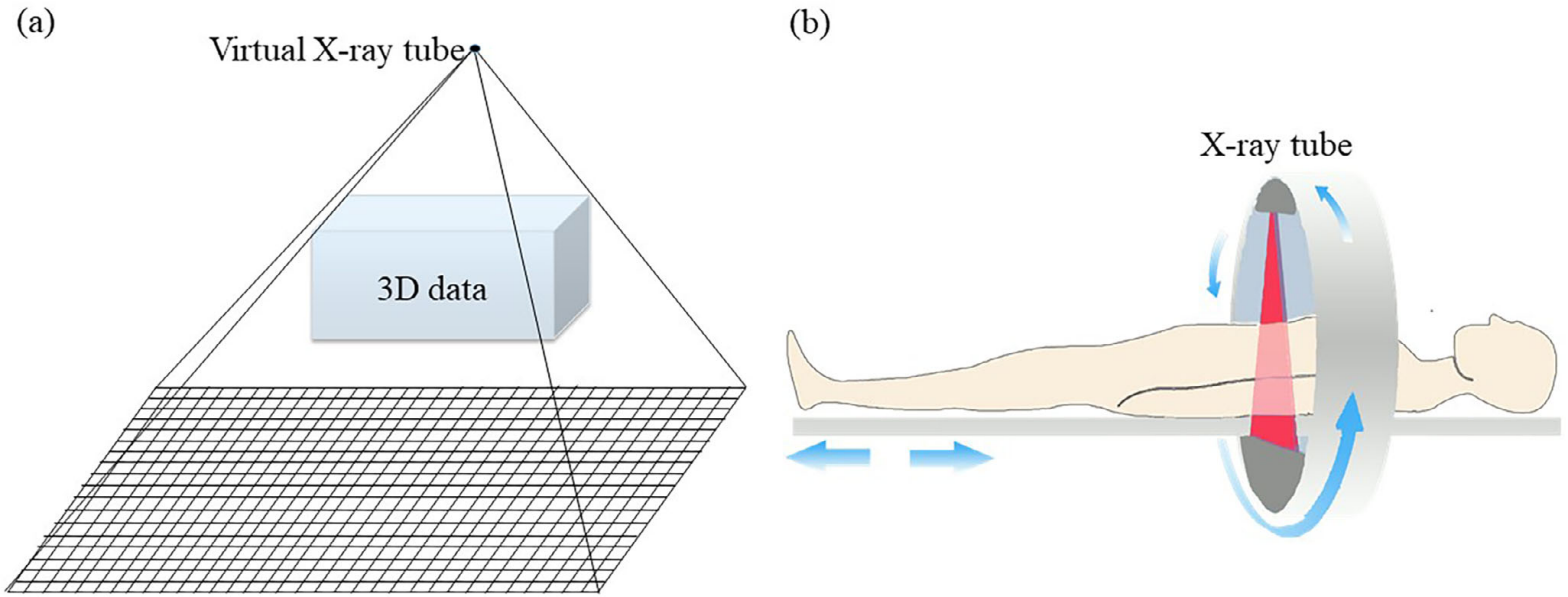
Schematic diagram of DRR generation versus actual topogram acquisition. (a) cDRR generation using a fixed virtual source. (b) Actual topogram acquisition with continuous table movement, which our uDRR method aims to simulate.

Conventional DRR (cDRR) generation uses a single, fixed virtual X‐ray source to project rays through the entire CT volume (Figure 1a).^19^ This static model does not accurately represent the actual topogram acquisition, which involves continuous table movement. To better emulate the physical scanning geometry, we propose a method that utilizes multiple virtual source points to generate local DRRs, producing a more realistic DRR, termed uDRR (Figure 1b). Virtual source points were placed along the z‑axis at intervals of 0.69 mm, matching the pixel spacing of the generated topograms. The number of virtual source positions is therefore determined by the z‑extent of each CT volume divided by 0.69 mm. Key geometric parameters (source‐to‐isocenter and source‐to‐detector distances) were configured based on actual CT scanner settings extracted from DICOM tags. The synthetic topograms were generated with a pixel spacing of 0.69 × 0.69 mm and a horizontal matrix of 768 pixels, consistent with typical clinical resolutions.

#### 2.1.2 Domain adaptation via CycleGAN

**FIGURE 2:**
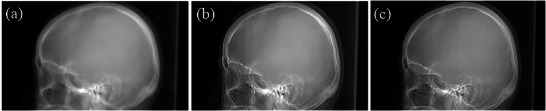
Comparison of generated images. (a) uDRR. (b) uTopogram (style‐transferred from uDRR). (c) Real topogram (ground truth). The mean absolute error (MAE) between uDRR and the real topogram was 9.5 ±  6.2 HU, while the MAE between uTopogram and the real topogram was reduced to 9.0  ±  4.9 HU, confirming the effectiveness of the CycleGAN‑based domain adaptation in reducing pixel‑wise intensity discrepancies.

While DRRs and real topograms share structural similarities, discrepancies in pixel intensity and texture persist (Figure 2). To bridge this domain gap, we employed a Cycle‐Consistent Adversarial Network (CycleGAN)^20^ to translate DRRs into the style of real topograms, creating synthetic topograms. The CycleGAN was trained in an unpaired manner on 100 real topograms and 100 randomly selected uDRRs. The stylized output of a cDRR is termed a cTopogram, and that of a uDRR is termed a uTopogram. In other words, cTopogram and uTopogram are the CycleGAN‐translated versions of cDRR and uDRR, respectively.

### 2.2 Eyeball mask generation network (EyeGen‐Net)

#### 2.2.1 Data preprocessing

For each synthetic topogram, the foreground region was identified via thresholding and binarization. A square Region of Interest (ROI) of 256 × 256 pixels (256 mm × 256 mm), centered on the image centroid, was extracted. Corresponding CT volumes were manually annotated by two senior radiologists using ITK‐SNAP (version 3.8). Annotation was performed slice‐by‐slice in the axial plane, defining the eyeball as the sclera–cornea boundary inclusive of the anterior chamber and lens. The two radiologists first independently segmented 20 cases to establish annotation guidelines, then jointly reviewed and refined all remaining segmentations in consensus. The inter‐observer Dice coefficient for the initial 20 cases was 0.95 ± 0.02, indicating excellent agreement. The resulting 3D binary masks assign a value of 1 to eyeball voxels and 0 to background. A cubic sub‐volume (256 mm × 256 mm × 256 mm) corresponding to the topogram ROI was extracted from the 3D mask. To manage GPU memory, this sub‐volume was resampled to an isotropic resolution of 2.0 mm, resulting in a final gold standard mask size of 128^3^ voxels.

#### 2.2.2 Network architecture

**FIGURE 3:**
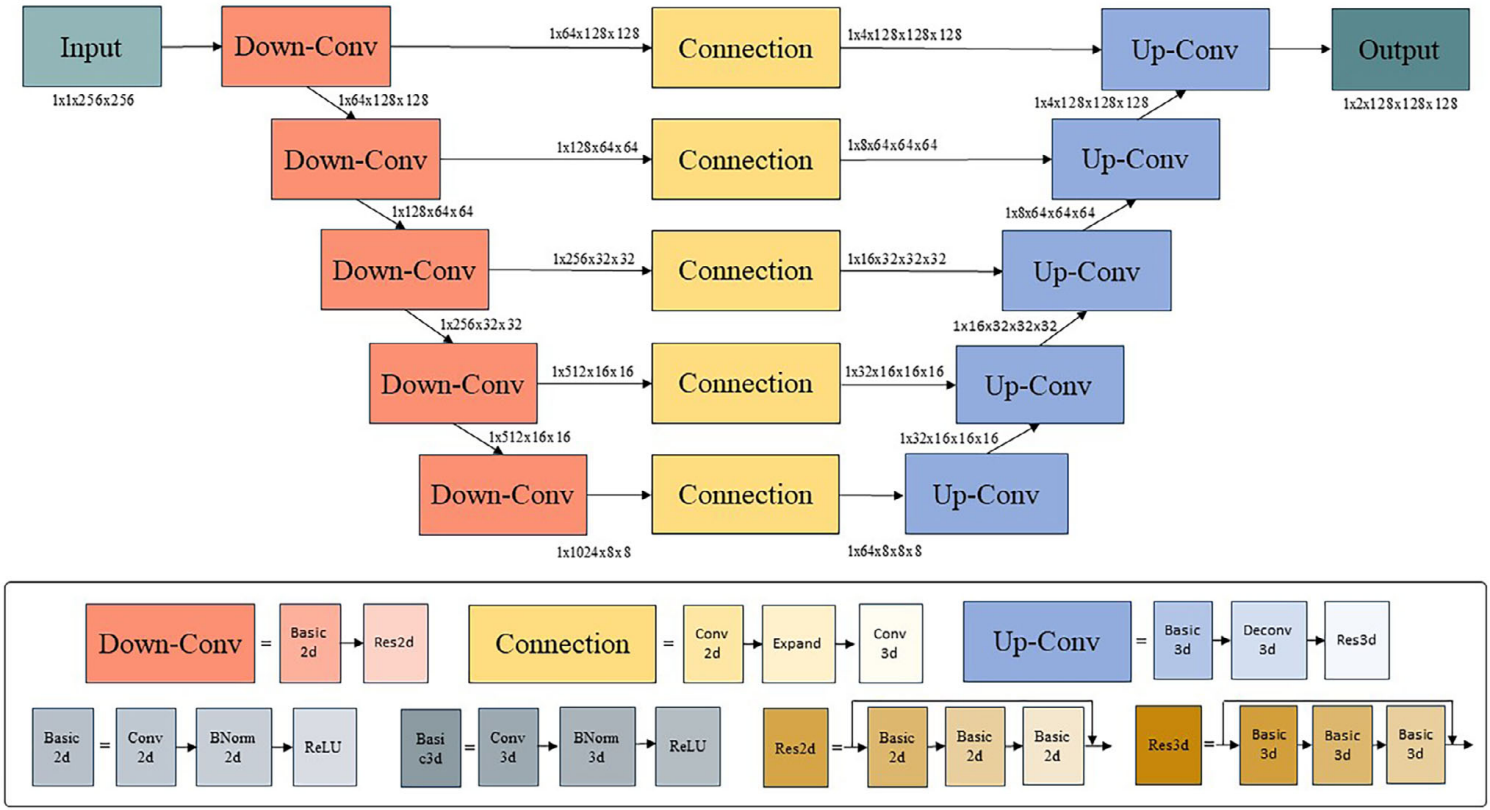
Architecture of the proposed EyeGen‐Net for 3D eyeball mask generation from a 2D topogram. Conv, Convolution. Deconv, Deconvolution.

The proposed EyeGen‐Net is illustrated in Figure 3. It takes a 2D topogram as input. The encoder uses 2D convolutional layers (stride = 2) with Batch Normalization (BN), ReLU activation, and residual connections for feature extraction. The decoder utilizes 3D transposed convolutional layers (stride = 2) with BN, ReLU, and residual connections for upsampling. At each corresponding resolution level, 2D feature maps from the encoder are replicated along the depth dimension and processed via 3D convolution, BN, and ReLU before being concatenated with the decoder features via skip connections. The final layer outputs a two‐channel 3D volume (128^3^ voxels per channel), representing the background and eyeball segmentation.

#### 2.2.3 Implementation details

The Dice Similarity Coefficient was used as the loss function.^21^ Optimization was performed using the AdamW algorithm^22^ with a learning rate of 1 × 10^−^^4^, *β*_1 _= 0.5, and *β*_2 _= 0.9. The model was implemented in PyTorch and trained for 600 epochs with a batch size of 16 on a Linux server equipped with an Intel Xeon Silver 4314 CPU and an NVIDIA A40 GPU. EyeGen‐Net was trained separately on cTopograms (baseline) and uTopograms (our proposed method) for comparison.

Statistical comparisons between the two models were performed on the 100 paired test samples. The normality of the pairwise differences (Baseline vs. Proposed) for DSC, HD, and ASD was first assessed using the Shapiro‐Wilk test. For metrics with normally distributed differences, a paired *t*‐test was applied; for metrics with non‐normally distributed differences, the Wilcoxon signed‐rank test was adopted. A two‐sided *p*‐value < 0.05 was considered statistically significant.

### 2.3 Phantom validation for eye protection

#### 2.3.1 Protection zone calculation

**FIGURE 4:**
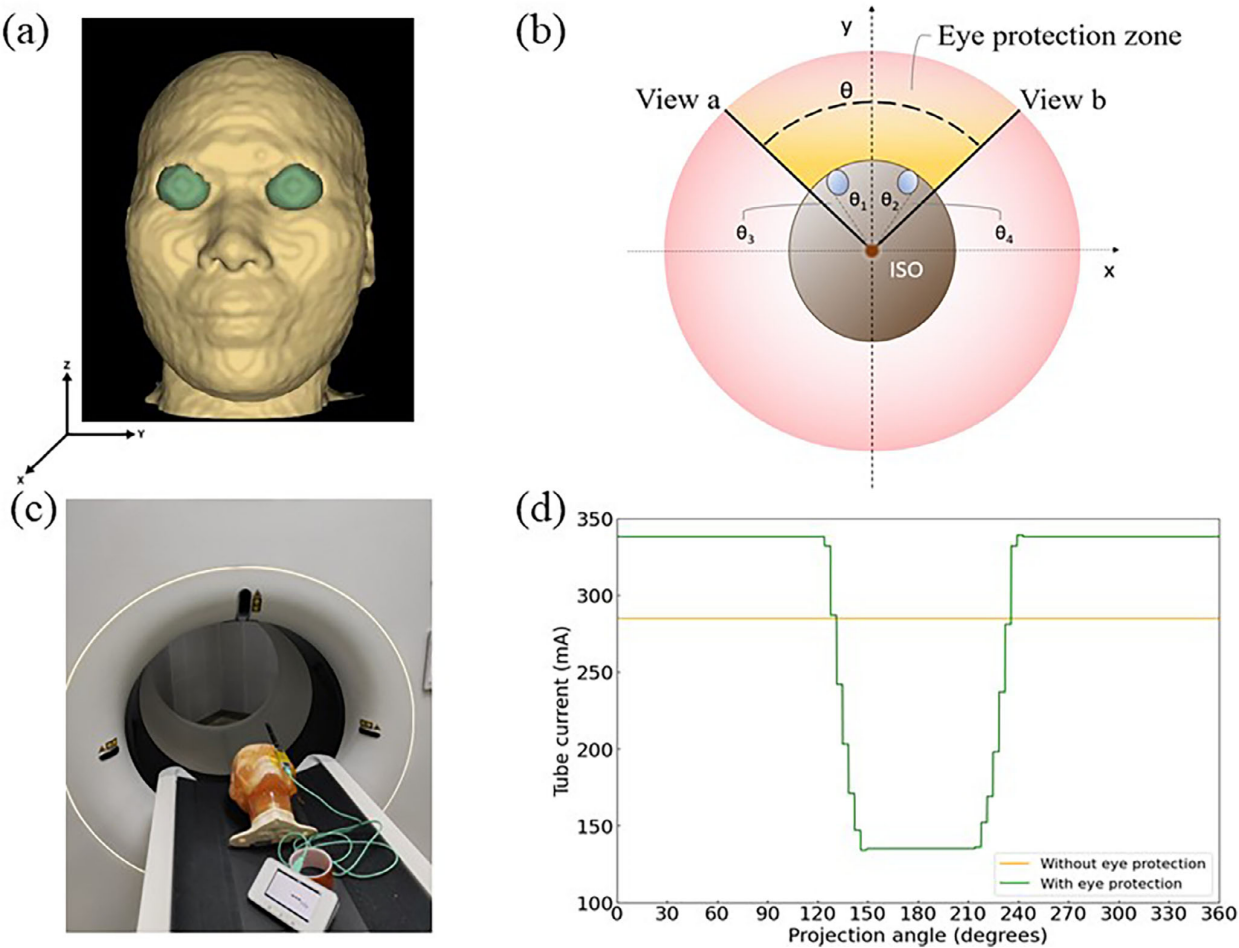
Illustration of eye protection and phantom verification. (a) The derived 3D eyeball mask generated by our EyeGen‐Net model is shown in green. (b) Schematic diagram for protection angle (θ) calculation from the scanner isocenter (ISO). The red circle represents the axial scanning plane; the brown circle represents the outer contour of the head; and the two blue circles represent the transverse cross‐sections of the left and right eyeballs. (c) Experimental setup for phantom validation. The PBU‑60 head phantom is positioned on the CT table with the RaySafe X2 dosimeter probe affixed to the eye surface for lens dose measurement. The same phantom setup was used for image quality evaluation under identical scanning conditions. (d) Representative tube current modulation curves recorded during the phantom experiment, comparing the standard (without eye protection) and OBTCM‑enabled (with eye protection) modes.

The derived 3D eyeball mask and scanner isocenter (ISO) were used to geometrically derive the protection parameters. The protection range was defined by the *Z*‐axis extent of the mask (Figure 4a). The protection angle (θ) was calculated by determining the outer tangents from the ISO to the left and right eyeball mask (angles *θ*_1_, *θ*_2_). A configurable redundancy margin (*θ*_3_, *θ*_4_ = 8° each) was added to ensure robust coverage. This margin was conservatively derived from the model's segmentation accuracy (ASD = 1.84 ± 0.65 mm), accounting for anatomical variations and isocenter alignment uncertainty (see Supplementary Materials for detailed derivation). The total protection angle is then defined as *θ* = *θ*_1_ + *θ*_2_ + *θ*_3_ + *θ*_4_ (Figure 4b). This angular region constitutes the eye protection zone, where tube current is reduced. The total radiation dose is preserved by proportionally increasing the tube current in the non‐protection zone to maintain global image quality.

#### 2.3.2 Dosimetry and image quality assessment

A PBU‐60 CT Whole Body Phantom (Kyoto Kagaku, Japan) was used for experimental validation. A calibrated dosimeter (RaySafe X2, Unfors Instruments) was placed on the phantom's eye surface to record the surface dose readings. All experiments were performed on a United Imaging uCT SiriuX scanner. Scans were conducted with and without the eye protection function enabled (Figure 4c, d). Both standard Axial and Helical scanning protocols were used to scan the eye region at tube voltages of 100, 120, and 140 kV. The target CT Dose Index volume (CTDIvol) was 46 mGy for the axial protocol and 42 mGy for the helical protocol, which are within the typical range for adult head CT examinations at our institution. The total tube current–time product (mAs) was held constant between the scans without and with eye protection: in the protected mode, the tube current was reduced within the protection zone and correspondingly increased outside the protection zone to maintain equivalent total mAs. The mAs values for each kV setting are reported in Table S2. The Helical protocol used a pitch of 0.5875. All scans employed a 1 mm slice thickness and were reconstructed using the Filtered Back Projection (FBP) algorithm. Critically, all post‐processing and image‐based denoising filters (including any AI‐driven noise reduction) were disabled for both scanning conditions to ensure a fair and intrinsic comparison of image quality.

To assess image quality, we evaluated image noise by measuring the standard deviation of Hounsfield Units (HU) within two elliptical Regions of Interest (ROIs) placed in homogeneous soft‑tissue regions of the brain, with areas of 441.9 mm^2^ and 412.9 mm^2^ (Figure 7). For each scanning condition (with and without eye protection), ROIs were carefully positioned in the same locations across all images to ensure consistency. The mean noise value from the two ROIs was calculated for each image, and the values with and without protection were compared. To further validate the effect of OBTCM on high‐contrast spatial resolution and low‐contrast detectability, a Catphan 700 phantom (The Phantom Laboratory, Salem, NY) was scanned using the identical axial and helical protocols. High‐contrast spatial resolution was evaluated using the high‐resolution module, and low‐contrast detectability was evaluated using the low‐contrast module. Measurements were performed with and without eye protection for direct comparison.

## 3 RESULTS

### 3.1 Data simulation performance

**FIGURE 5:**
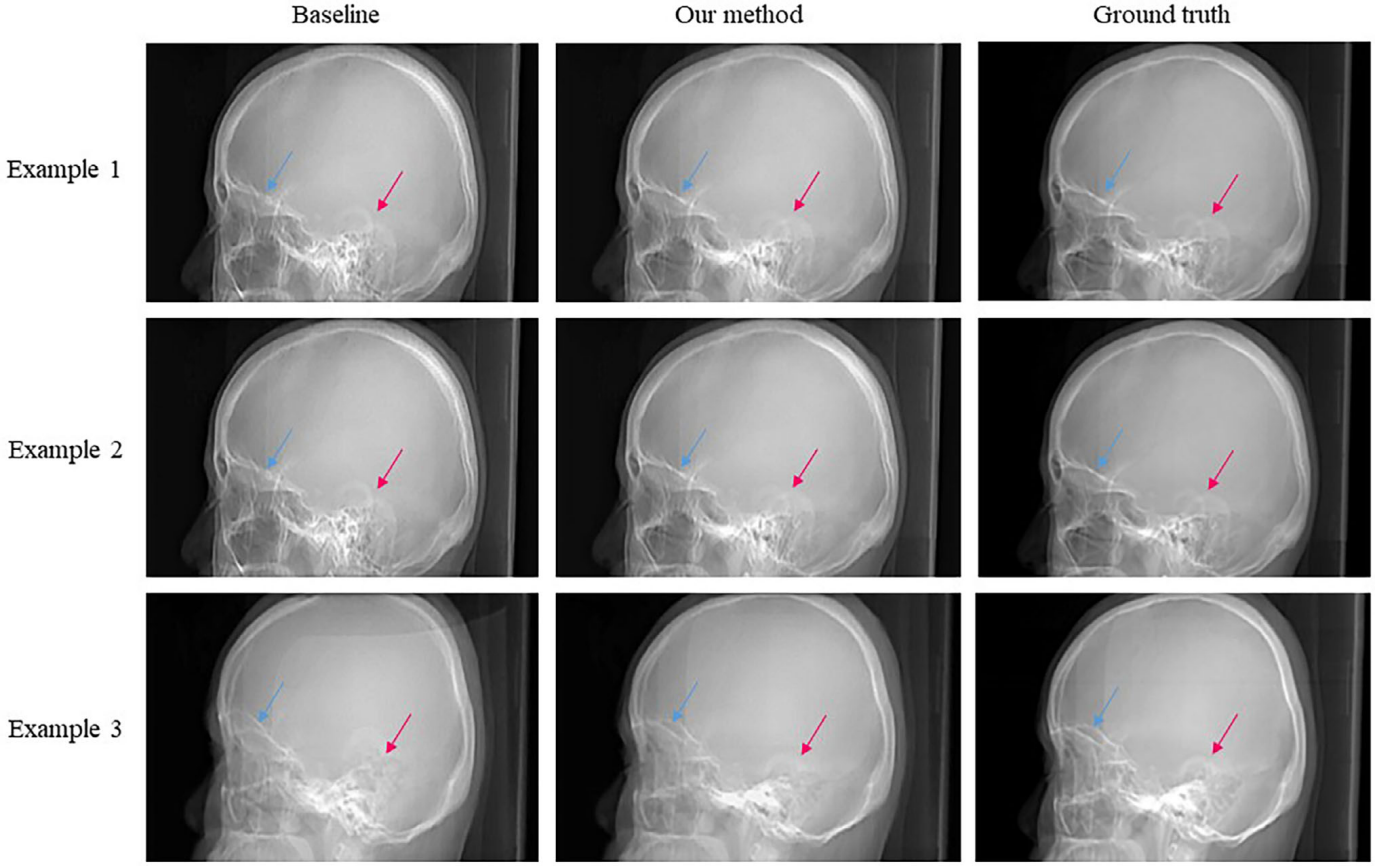
Qualitative comparison of synthetic topograms. Representative examples showing the baseline cTopogram (Baseline), the proposed uTopogram (Our Method), and the real topogram (Ground Truth). Blue arrows indicate blurred orbital rim margins in the cTopogram; red arrows indicate morphological distortion of auricular structures in the cTopogram.

Figure 5 shows representative cTopograms and uTopograms compared to a real topogram. The cTopogram exhibits noticeable artifacts, including blurred orbital rim margins (blue arrows) and morphological distortion of auricular structures (red arrows). In contrast, the uTopogram demonstrates superior preservation of anatomical detail, enhanced structural consistency with the ground truth, and overall more realistic synthetic image quality.

### 3.2 Eyeball mask generation performance

**FIGURE 6:**
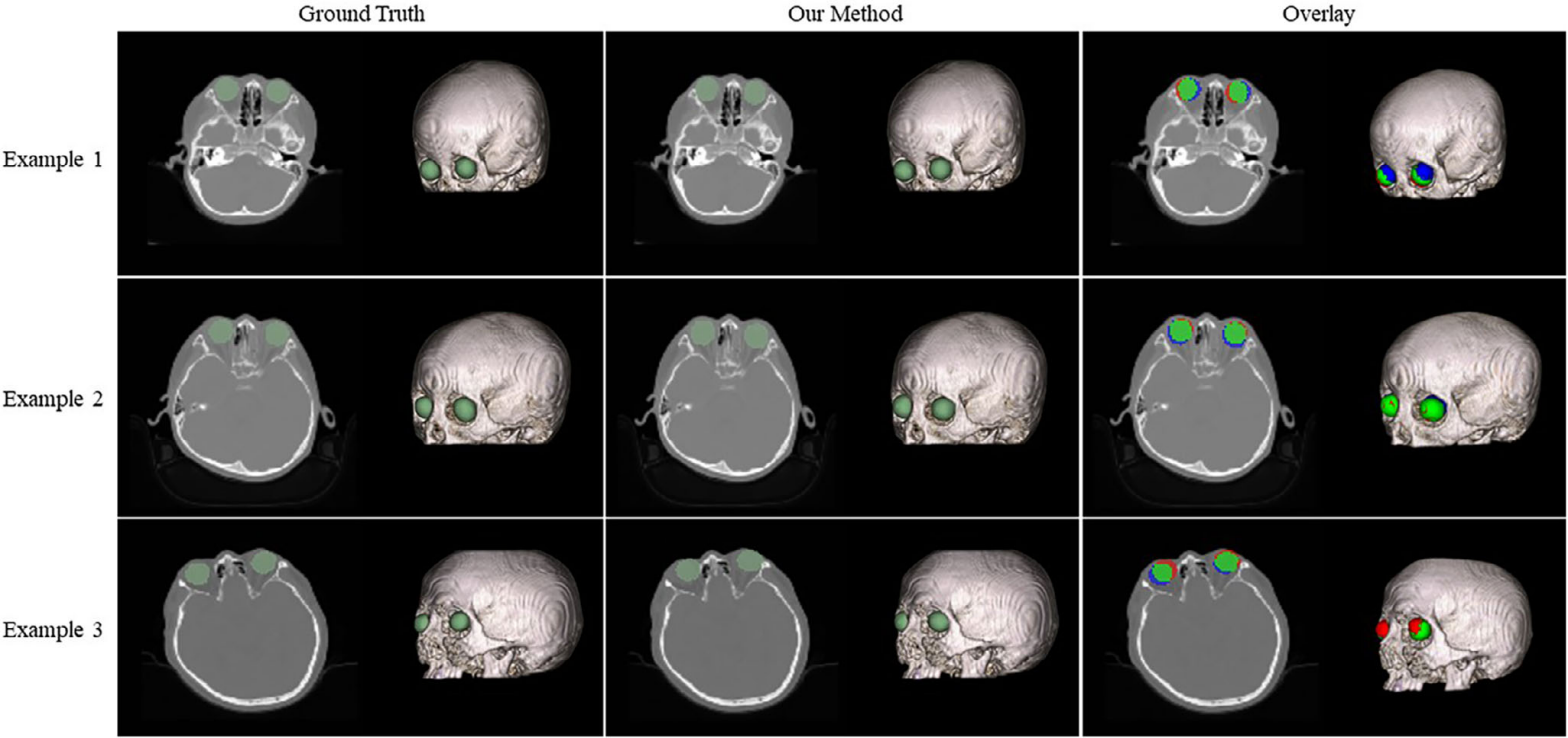
Sample results of 3D eyeball model generation. For each example, the ground truth mask (left), the predicted mask from our method (middle), and the color‐coded overlay (right) are shown. Each mask is displayed in axial view and volume rendering. In the overlay: green indicates voxels where the prediction and ground truth agree (true positive); red indicates voxels predicted as eyeball but not present in the ground truth (false positive); blue indicates voxels present in the ground truth but missed by the prediction (false negative).

The quantitative comparison between the baseline model (trained on cTopograms) and our proposed method (trained on uTopograms) is summarized in Table 1. Our method achieved higher segmentation accuracy on the test set. The Dice Similarity Coefficient (DSC) improved from 0.78 ± 0.09 to 0.79 ± 0.08 (*p* = 0.09). The Hausdorff Distance (HD) was reduced from 5.67 ± 1.68 mm to 5.40 ± 1.57 mm (*p* = 0.01), representing a statistically significant improvement. The Average Surface Distance (ASD) decreased from 1.90 ± 0.68 mm to 1.84 ± 0.65 mm (*p* = 0.09). All three metrics moved in a positive direction, and the lower standard deviations indicate improved stability. Notably, our model, using only a single‐view topogram, surpassed the reported DSC performance (0.71) of a method utilizing two‐view topograms for organ localization.^17^ Qualitative assessment in Figure 6 shows that the predicted masks exhibit highly consistent global shapes and smooth contours compared to the ground truth.

**TABLE 1:** Quantitative comparison of eyeball mask generation performance (mean ± standard deviation).

Model	DSC	HD (mm)	ASD (mm)
Baseline	0.78 ± 0.09	5.67 ± 1.68	1.90 ± 0.68
Ours	0.79 ± 0.08	5.40 ± 1.57	1.84 ± 0.65

*Note*: Paired statistical comparisons (*t*‐test or Wilcoxon signed‐rank depending on Shapiro‐Wilk normality test): DSC *p* = 0.09; HD *p* = 0.01; ASD *p* = 0.09.

### 3.3 Eye protection performance

The dose measurement results are presented in Table 2. Enabling the eye protection function resulted in an average dose reduction of approximately 30% in axial scanning mode across all tube voltages. In helical mode, the dose reduction also reached up to ∼30%, with minor variations attributable to the non‐fixed starting angle of the X‐ray tube.

**TABLE 2:** RaySafe X2 readings at the eye surface without and with eye protection.

Acquisition mode	Tube voltage (kV)	Dose without eye protection (mGy)	Dose with eye protection (mGy)	Change in dose (%)
Axial	100	4.39	3.07	30.07
	120	4.38	3.05	30.37
	140	4.34	3.02	30.46
Helical	100	13.39	10.05	24.93
	120	14.10	9.74	30.91
	140	14.81	10.74	27.50

**FIGURE 7:**
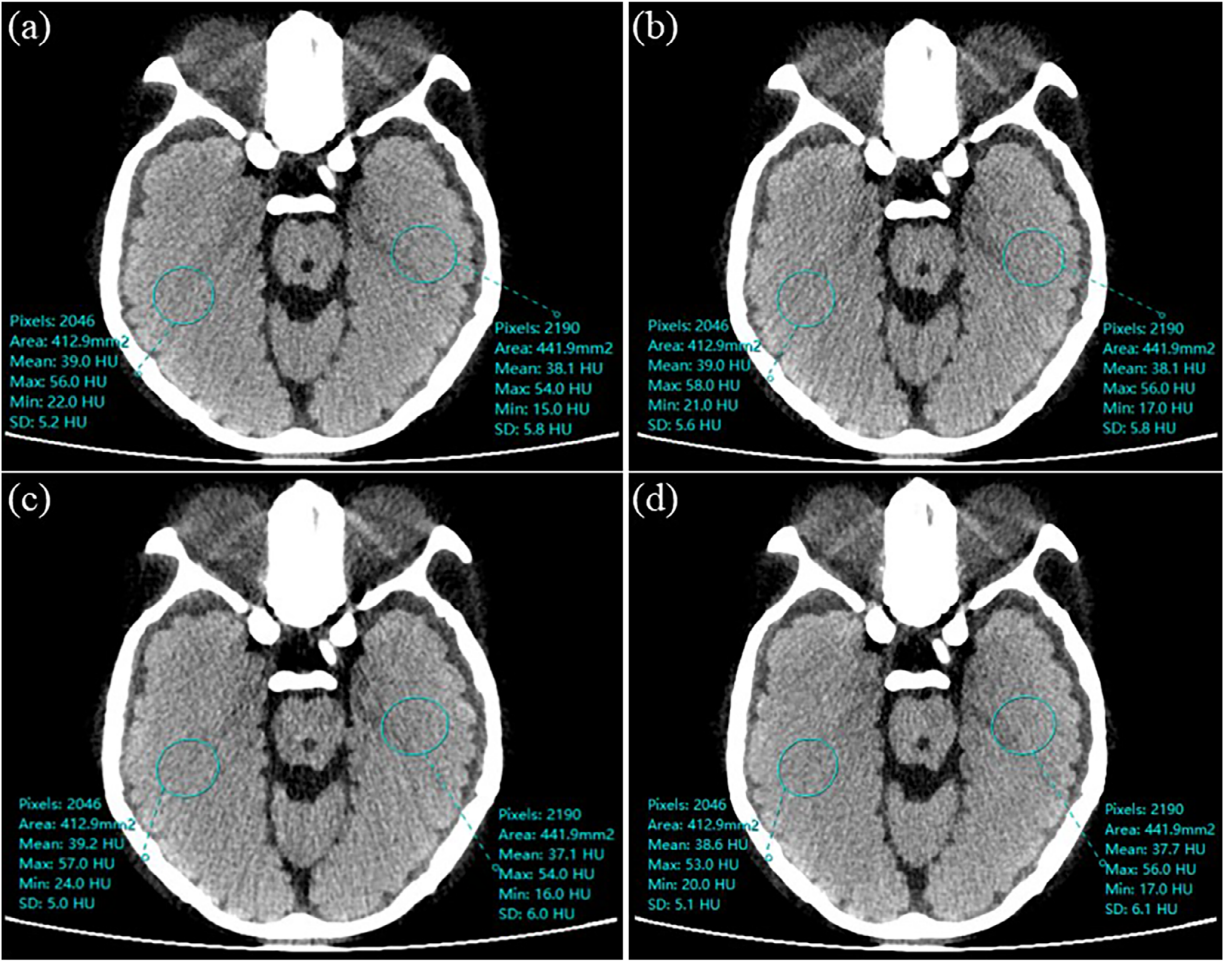
Exemplary phantom images and noise measurement ROIs (blue circles). (a) Axial scan without eye protection. (b) Axial scan with eye protection. (c) Helical scan without eye protection. (d) Helical scan with eye protection. For each ROI, the annotated values report the number of pixels, area, mean CT number, maximum, minimum, and standard deviation (SD). The SD directly corresponds to the noise used in the quantitative analysis (Table 3).

Image quality assessment results are summarized in Table 3. The noise levels (standard deviation in HU) remained consistent, with a maximum deviation of 13.46%, which falls within the accepted clinical tolerance (≤15%) per standards like IEC 61223‐3‐5.^23^ This demonstrates that the dose “saved” over the eyes was effectively redistributed to other angles to maintain the overall signal‐to‐noise performance. Beyond noise evaluation, the Catphan 700 phantom assessment confirmed that the eye‐protecting OBTCM had no measurable impact on high‐contrast spatial resolution (7 vs. 7 lp/cm) or low‐contrast detectability (7 vs. 7 visible targets at the 1.0% contrast level). Complete Catphan results are provided in Figures S1, S2. Example images are shown in Figure 7.

**TABLE 3:** Image quality comparison without and with eye protection.

Acquisition mode	Tube voltage (kV)	ROI	Noise without eye protection (HU)	Noise with eye protection (HU)	Change in noise (%)
Axial	100	ROI1	7.7	7.8	1.30%
	100	ROI2	6.4	6.5	1.56%
	120	ROI1	5.8	5.8	0.00%
	120	ROI2	5.2	5.6	7.69%
	140	ROI1	5.3	5.8	9.43%
Helical	140	ROI2	5.1	5.5	7.84%
	100	ROI1	5.9	6.4	8.47%
	100	ROI2	5.5	6.1	10.91%
	120	ROI1	6.0	6.1	1.67%
	120	ROI2	5.0	5.1	2.00%
	140	ROI1	5.2	5.9	13.46%
	140	ROI2	5.6	5.7	1.79%

## 4 DISCUSSION

### 4.1 Principal findings

This study developed and validated a novel deep learning framework for generating an accurate 3D eyeball model from a routine single‐view topogram. Our advanced data simulation pipeline produced highly realistic training data (uTopograms), leading to an EyeGen‐Net model that achieved robust segmentation performance (DSC: 0.79). Most importantly, phantom validation confirmed the clinical utility of this approach, demonstrating that the derived model enables OBTCM capable of delivering a substantial ∼30% reduction in lens dose while preserving diagnostic image quality.

### 4.2 Comparison with existing techniques

Our work addresses limitations inherent in current OBTCM strategies. Compared to fixed‐angle methods,^13^, ^14^, ^15^ our personalized approach dynamically defines the protection zone based on actual patient anatomy. This avoids the two main pitfalls of fixed arcs: 1) incomplete protection for patients whose ocular anatomy places the lens outside the standard fixed arc, and 2) unnecessary radiation dose and potential image quality degradation to anterior non‐ocular tissues in patients whose lenses are well within the arc.

Compared to dual‐view, synthetic‐CT‐based methods,^16^, ^17^, ^18^ our solution offers greater clinical practicality. By requiring only a single‐view topogram—the default in most head CT protocols—it eliminates the need for an additional lateral scan, streamlining workflow. Furthermore, we provide a complete technical disclosure specifically for small organ (eyeball) segmentation, a detail often omitted in prior literature. While dual‐view methods may theoretically provide more 3D context, our results show that a specialized single‐view network can achieve comparable or superior segmentation accuracy (e.g., DSC of 0.79 vs. 0.71 reported in [17]).

### 4.3 Clinical implications and translation

The primary clinical implication is the enabling of true patient‐specific eye protection that aligns with the ALARA principle. The proposed pipeline is designed for seamless integration into routine clinical head CT workflows without requiring additional user interaction. After the patient is positioned and the single‐view topogram is acquired, the trained EyeGen‐Net model—deployed directly on the scanner console—automatically generates the 3D eyeball mask in under one second. The protection angle and *z*‐axis coverage are then calculated geometrically and immediately pushed to the scanner's real‐time tube current modulation module. The entire computational step introduces negligible delay before the diagnostic scan commences. Because only a single‐view topogram is required, no additional scout acquisitions or changes to standard clinical protocols are needed, facilitating straightforward adoption of personalized OBTCM.

The measured 30% dose reduction is clinically significant, directly contributing to meeting the stringent ICRP dose limit for the lens.^10^ The preservation of image quality, as evidenced in our phantom study, is crucial for maintaining diagnostic confidence, addressing a key concern when modulating tube current.

### 4.4 Limitations

This study has several limitations. First, the model was developed and validated using a combination of synthetic data and a single‐center clinical dataset. While our simulation pipeline mitigates data scarcity, a multicenter study with a larger, more diverse patient population is necessary to fully establish generalizability. Second, the current framework is designed specifically for the eyeballs. Expanding it to include other radiosensitive organs (e.g., thyroid, breast) would maximize its clinical impact. Third, while our phantom evaluations confirmed no measurable degradation in image noise, high‐contrast spatial resolution, or low‐contrast detectability, a more comprehensive assessment of diagnostic image quality under OBTCM—including systematic evaluation of potential local artifacts—is warranted in future clinical studies. Fourth, the dosimetric measurements relied on RaySafe X2 detector readings, which represent air kerma at the eye surface rather than absolute absorbed dose at the lens. Backscatter, measurement point offset, and beam quality corrections were not applied. As our primary endpoint is relative dose reduction, these multiplicative factors do not affect the reported percentage. Future studies using optically stimulated luminescence dosimeters would enable absolute lens dose quantification.

### 4.5 Future work

Future directions include: 1) Conducting a multicenter trial to validate robustness across different scanner models and patient demographics; 2) Extending the network architecture to simultaneously segment multiple organs from a single topogram; and 3) Performing a clinical reader study to objectively assess the impact on diagnostic performance.

## 5 CONCLUSION

This study presents a practical and effective deep learning solution for personalized eye protection in head CT. By accurately generating a 3D eyeball model directly from a single‐view topogram, our work bridges the gap between the concept of patient‐specific OBTCM and routine clinical practice. The validated pipeline—comprising an advanced data simulation method, a dedicated generative network, and a clear pathway for clinical integration—enables substantial lens dose reduction without compromising image quality. This represents a significant step forward in personalized radiology and patient safety.

## AUTHOR CONTRIBUTIONS

All authors agreed on the content of the study. MXL and ZL designed the study, performed the experiments, analyzed the data, and wrote the manuscript. CSH performed the experiments, analyzed the data. LMH provided research guidance, reviewed and revised the manuscript. WY supervised the overall research, collected all data for this study, made the mask annotations, and revised the manuscript. All authors read and approved the final manuscript.

## CONFLICT OF INTEREST STATEMENT

The authors declare no competing interests.

## ETHICS STATEMENT

This retrospective study was conducted in accordance with the ethical principles of the Declaration of Helsinki. The protocol for using the anonymized dataset was reviewed and approved by the Institutional Review Board (IRB) of Zhujiang Hospital, Southern Medical University (Approval No.: 2026‐KY‐030‐01). Owing to the retrospective design and the use of fully anonymized data, the requirement for obtaining written informed consent was waived by the aforementioned IRB.

## Supporting information




**Supporting Information**: acm270665‐sup‐0001‐SuppMat.docx

## Data Availability

Data are available from the corresponding author upon reasonable request.

## References

[1] Tian X , Li X , Segars WP , et al. Prospective optimization of CT under tube current modulation: i. organ dose. Spie Medical Imaging. 2014;9033.

[2] Yuan MK , Tsai DC , Chang SC , et al. The risk of cataract associated with repeated head and neck CT studies: a nationwide population‐based study. Am J Roentgenol. 2013;201(3):626‐630. 23971456 10.2214/AJR.12.9652

[3] Mohammadpour M , Movahedan ZE , Jabbarvand M , Hashemi H . Radiation cataract: clinicopathologic report. J Cataract Refract Surg. 2013;39(2):285‐288. 23332255 10.1016/j.jcrs.2012.11.016

[4] Peled A , Moshe S , Chodick G . [Ionizing radiation and the risk for cataract and lens opacities]. Harefuah. 2018;157(10):650‐654. 30343544

[5] Buchberger B , Scholl K , Krabbe L , et al. Radiation exposure by medical X‐ray applications. Ger Med Sci. 2022;20:Doc06. 35465642 10.3205/000308 PMC9006309

[6] Cao CF , Ma KL , Shan H , et al. CT scans and cancer risks: a systematic review and dose‐response meta‐analysis. BMC Cancer. 2022;22(1):1238. 36451138 10.1186/s12885-022-10310-2 PMC9710150

[7] Berrington de Gonzalez A , Pasqual E , Veiga L . Epidemiological studies of CT scans and cancer risk: the state of the science. Br J Radiol. 2021;94(1126):20210471. 34545766 10.1259/bjr.20210471 PMC9328069

[8] Bosch de Basea Gomez M , Thierry‐Chef I , Harbron R , et al. Risk of hematological malignancies from CT radiation exposure in children, adolescents and young adults. Nat Med. 2023;29(12):3111‐3119. 37946058 10.1038/s41591-023-02620-0 PMC10719096

[9] Frush DP , Frija G , Allen B , et al. CT radiation exposure and cancer risk: from knowing to acting. Pediatr Radiol. 2024;54(8):1407‐1409. 38750325 10.1007/s00247-024-05949-x

[10] Stewart FA , Akleyev AV , Hauer‐Jensen M , et al. ICRP publication 118: iCRP statement on tissue reactions and early and late effects of radiation in normal tissues and organs–threshold doses for tissue reactions in a radiation protection context. Ann ICRP. 2012;41(1‐2):1‐322. 10.1016/j.icrp.2012.02.001 22925378

[11] Ketelsen D , Buchgeister M , Fenchel M , et al. Automated computed tomography dose‐saving algorithm to protect radiosensitive tissues: estimation of radiation exposure and image quality considerations. Invest Radiol. 2012;47(2):148‐152. 21934513 10.1097/RLI.0b013e3182311504

[12] Rekdal AL , Radiation dose and image quality in CT: comparison and evaluation of two different organ‐based tube current modulation techniques and their impact on organ dose and image noise [master's thesis]. Norwegian University of Science and Technology; 2019.

[13] Kim JS , Kwon SM , Kim JM , et al. New organ‐based tube current modulation method to reduce the radiation dose during computed tomography of the head: evaluation of image quality and radiation dose to the eyes in the phantom study. Radiol Med. 2017;122:601‐608. 28341967 10.1007/s11547-017-0755-5

[14] Duan X , Wang J , Christner JA , Leng S , Grant KL , McCollough CH . Dose reduction to anterior surfaces with organ‐based tube‐current modulation: evaluation of performance in a phantom study. Am J Roentgenol. 2011;197:689‐695. 21862813 10.2214/AJR.10.6061

[15] Fu W , Sturgeon GM , Agasthya G , et al. Estimation of breast dose reduction potential for organ‐based tube current modulated CT with wide dose reduction arc. In: Proceedings of SPIE Medical Imaging. 2017;10132 doi:10.1117/12.2255797

[16] Kachelrieß M . Risikominimierende Röhrenstrommodulation in der Computertomographie [Risk‐minimizing tube current modulation for computed tomography]. Radiologie. 2023;63(7):523‐529. 37306750 10.1007/s00117-023-01160-5

[17] Liu C , Klein L , Huang Y , et al. Two‐view topogram‐based anatomy‐guided CT reconstruction for prospective risk minimization. Sci Rep. 2024;14(1):12945. 38653993 10.1038/s41598-024-59731-y PMC11039459

[18] Klein L , Liu C , Steidel J , et al. Patient‐specific radiation risk‐based tube current modulation for diagnostic CT. Med Phys. 2022;49(7):4391‐4403. 35421263 10.1002/mp.15673

[19] Milickovic N , Baltas D , Giannouli S , et al. CT imaging based digitally reconstructed radiographs and their application in brachytherapy. Phys Med Biol. 2000;45(10):2787‐2800. 11049172 10.1088/0031-9155/45/10/305

[20] Zhu JY , Park T , Isola P , Efros AA , Unpaired image‐to‐image translation using cycle‐consistent adversarial networks. In: Proc IEEE Int Conf Comput Vis. 2017:2223‐2232.

[21] Milletari F , Navab N , Ahmadi SA . V‐Net: fully convolutional neural networks for volumetric medical image segmentation. 2016 Fourth Int Conf 3D Vis (3DV). IEEE; 2016:565‐571.

[22] Loshchilov I , Hutter F , Decoupled weight decay regularization. arXiv preprint arXiv:1711.05101. 2017.

[23] International Electrotechnical Commission. IEC 61223‐3‐5: Evaluation and routine testing in medical imaging departments—Part 3–5: Acceptance and constancy tests—Imaging performance of computed tomography X‐ray equipment. 2019.

