# Supplementary material for: Personalized eye protection for head CT organ‐based tube current modulation: A deep learning approach to derive 3D eyeball models from a single‐view topogram
Source: J Appl Clin Med Phys. 2026 Jun 23;27(7):e70665. doi: 10.1002/acm2.70665 (PMC13291186; doi:10.1002/acm2.70665)
Supplement: Supplementary file 1 — Supporting Information: acm270665‐sup‐0001‐SuppMat.docx [file ACM2-27-e70665-s001.docx]

**Personalized Eye Protection for Head CT Organ-Based Tube Current Modulation: A Deep Learning Approach to Derive 3D Eyeball Models from a Single-View Topogram**

Index of supplementary materials

[Supplementary Materials: Derivation of Redundancy Margins θ₃ and θ₄ 2](#_Toc229348684)

[Supplementary Materials: TABLES S1-S2 5](#_Toc229348685)

[Supplementary Materials: FIGURES S1-S2 6](#_Toc229348686)

# Supplementary Materials: Derivation of Redundancy Margins θ₃ and θ₄

The redundancy margins θ₃ and θ₄ were set to 8° based on a conservative estimate derived from the segmentation accuracy of the EyeGen-Net model, combined with anatomical considerations and practical safety margins.

**S1. Model Segmentation Accuracy**

As reported in Table 1 of the main manuscript, the EyeGen-Net model achieved an Average Surface Distance (ASD) of **1.84 ± 0.65 mm.** To ensure robust coverage of the eyeball despite segmentation uncertainty, we adopted a highly conservative upper bound by taking the **mean ASD plus three standard deviations**:

**Error_upper = 1.84 mm + 3 × 0.65 mm = 3.79 mm**

This captures approximately 99.7% of expected boundary errors, providing a statistically rigorous foundation for the safety margin.

**S2. Ideal Geometric Calculation**

Under ideal conditions where the scanner isocenter is perfectly aligned with the center of the head:

- Typical adult head radius ≈ **80 mm**
- Eyeball radius ≈ **12 mm**
- Therefore, the distance from the isocenter to the center of the eyeball ≈ 80 mm - 12 mm = **68 mm**

Under this ideal geometry, the angular uncertainty corresponding to the 3.79 mm error would be:

**θ_margin = arcsin (Error_upper / Distance) = arcsin (3.79 mm / 68 mm) = arcsin (0.0557) ≈ 3.2°**

**S3. Clinical Considerations Requiring a Larger Margin**

However, in clinical practice, several factors necessitate a more generous safety margin:

1. **Isocenter alignment uncertainty:** The patient’s head may not be perfectly centered within the scanning field, introducing additional geometric variability. This can effectively increase the distance from the isocenter to the eyeball or alter the angular relationship.
2. **Anatomical variation:** Pediatric patients have smaller head sizes. For example, a child with a head circumference of 34 cm has a head radius of approximately 54 mm. Using the same error estimate of 3.79 mm, the angular uncertainty would be:

θ_margin = arcsin (3.79 mm / (54 mm - 12 mm)) = arcsin (3.79 mm / 42 mm) = arcsin (0.0902) ≈ 5.2°

1. **Patient positioning and motion:**Minor variations in head positioning between scans can affect the effective protection angle.

**S4. Selection of 8° as a Robust Clinical Margin**

To account for these real-world factors and ensure reliable eye protection across a wide range of clinical scenarios, we conservatively set the redundancy margins to 8°. This

value:

- Is substantially larger than the ideal-case calculation of 3.2°.
- Provides ample buffer for isocenter misalignment and anatomical variation (e.g., the pediatric case yields 5.2°, which is still below 8°).
- Remains clinically practical without excessively compromising image quality, as validated by our phantom study demonstrating approximately 30% dose reduction with preserved image quality.

**S5. Validation**

The adequacy of the 8° margin is indirectly confirmed by the phantom study results (Table 2 and Table 3 in the main manuscript), which demonstrate that the eye protection function achieved substantial dose reduction (~30%) while maintaining image noise within clinically acceptable limits. If the margin were too small, the eye might occasionally fall outside the protected zone; if too large, unnecessary dose reduction would degrade image quality. The preserved image quality confirms that 8° strikes an appropriate balance.

# Supplementary Materials: TABLES S1-S2

TABLE S1 Statistics of eyeball angular positions (θ₁ and θ₂ as defined in Figure 4b) derived from 100 clinical cases.

| **Statistic** | **θ₁** **(°)** | **θ_2_** **(°)** | **θ₁+θ_2_** **(°)** |
| --- | --- | --- | --- |
| Mean ± SD | 33.2 ± 5.9 | 34.1 ± 5.4 | 67.3 ± 3.5 |
| Minimum | 12.4 | 19.2 | 60.3 |
| Maximum | 46.1 | 52.2 | 77.5 |

TABLE S2 Tube current–time product (mAs) values for axial and helical head CT protocols without and with eye protection at different tube voltages (100, 120, and 140 kV).

| **Acquisition mode** | **Tube voltage (kV)** | **Tube current-time**  **product (mAs)** | **CTDIvol (mGy)** |
| --- | --- | --- | --- |
| Axial | 100 | 462 | 46.00 |
|  | 120 | 285 | 46.00 |
|  | 140 | 193 | 46.00 |
| Helical | 100 | 513 | 42.00 |
|  | 120 | 320 | 42.00 |
|  | 140 | 207 | 42.00 |

# Supplementary Materials: FIGURES S1-S2


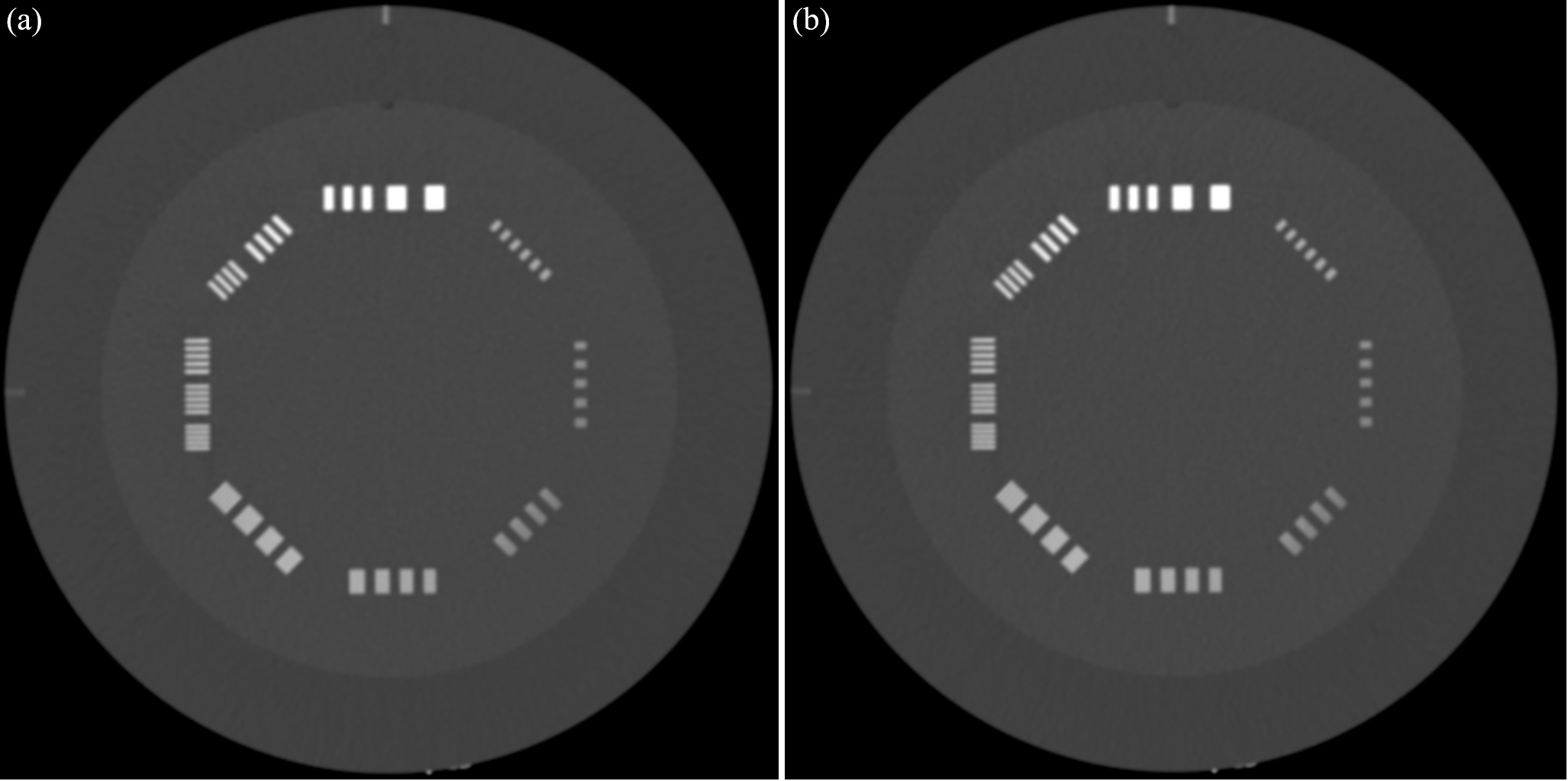


FIGURE S1 High-contrast spatial resolution assessment using the Catphan 700 High resolution module. (a) Scan without eye protection. (b) Scan with eye protection. The limiting spatial resolution was 7 lp/cm without eye protection and 7 lp/cm with eye protection, showing no measurable degradation from the OBTCM-enabled scan.


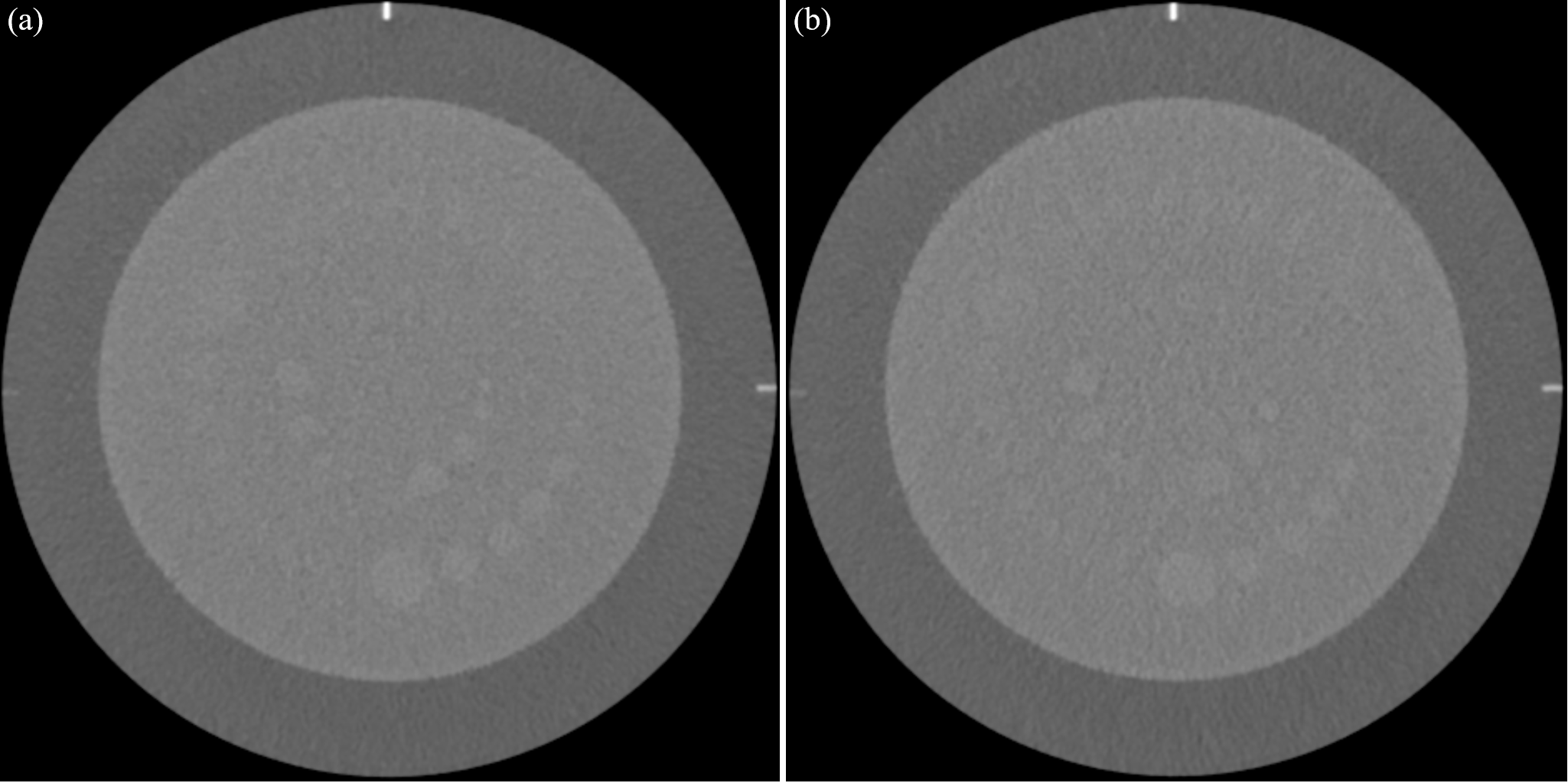


FIGURE S2 Low-contrast detectability assessment using the Catphan 700 low contrast module. (a) Scan without eye protection. (b) Scan with eye protection. The number of visible targets at the 1.0% nominal contrast level was 7 without eye protection and 7 with eye protection, showing no measurable degradation from the OBTCM-enabled scan.
